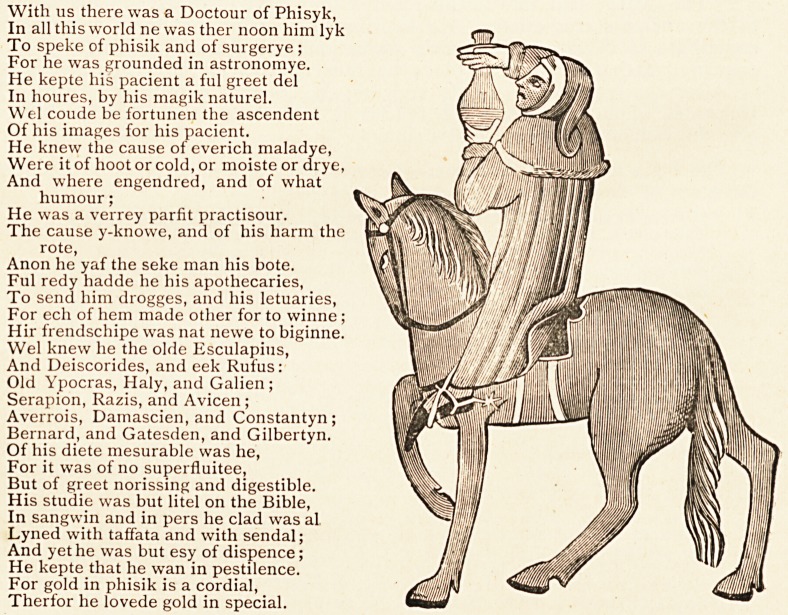# Scraps

**Published:** 1894-06

**Authors:** 


### SCRAPS

PICKED UP BY THE ASSISTANT-EDITOR.

---

**Clinical Records** (7).—According to a Melbourne correspondent of the *British Medical Journal* the very depressed state of their finances is responsible for the following: A lady who was suffering from an ailment peculiar to her sex called on a specialist for advice, and was asked by him why he had the honour of being selected by her, to which she replied that "just then she could not afford to consult a guinea-cologist, but that she had heard that he was only half-a-guinea-cologist!"

**English Abroad.**—In its account of the recent International Medical Congress the *Medical Record* says: The following is the report of Virchow's address at the inaugural session, as given in the *Official Journal* of the following day:

"The Professor Virchow, President of the last Congress, explaine the reasons that have decided the Physicians that in 1890 were reunited at Berlin, to choice Rom, as six of the XIth International Congress.

"The Orator said, that in this manner they have intended to make homage to the ancients traditions. He exprime the desire that the Congress, can contribute to elevatad the morals aspirations, the ties of the friendsip of all countries, so that the fraternal place that must approch all the civilited Nations."

After the conclusion of this address, the *Journal* continues: "Mrs. The Delegates whom names are the following, have taken the word in order, to give the welcome to their Compatriots."

**The "Fin de Siècle" Surgeon.**—I copy from the *Medical News* of May 26th three out of eleven verses signed "C. Rockwell":

If there's cause to suspect any ill effect produced by unseemly meals,
The modern M.D. doesn't wait, not he, to ask how his patient feels,
Nor to hear him state what he thinks he ate—but without any useless fuss
He lets down a string with a bucket-like thing, and samples his contents—thus

Into dark recesses which baffle guesses he pokes his 'scope with a grin,
Then turns on the light, and the luckless wight "all glorious is within."
When intestine strife makes a burden of life, and obstructionist lumps prevail,
Though the case be grave, to the surgeon brave there is no such word as fail.

With a wide incision and free division he charges upon the foe,
Takes what he thinks best, and puts back the rest in the place where it ought to go,
In a half-way measure he takes no pleasure, so fits out his man beside
With the latest in rings, nickel buttons, and springs, and sutures him up with pride.

**Medical Philology** (X.).—In the *Promptorium* is to be found "BONSCHAWE, sekeness. *Tessedo, sciasis.*" Pynson's printed edition (1499) gives it as "bonshawe." Mr. Way's note is:

> The baneschawe, *oscedo*. CATH. ANGL. "*Oscedo, quedam infirmitas quo ora infantium exulcerantur, i.e. oscitatio, oris apertio*, a boneshawe." ORT. "*De infirmitatibus.* Ba[illegible]eschaw, *cratica, i. passus.*" Roy. MS. 17 C. XVII., f. 40. John Arderne, who was surgeon to Edward III., says in his Chirurgica, "*ad guttam in osse, que dicitur* bonschawe, *multum valet oleum de vitellis ovorum, si inde ungatur.*" Sloan. MS. 56, f. 18b. In Sloan. MS. 100, f. 7, is given the recipe for "a good medicyn for boonschawe. Take bawme and fevirfoie, the oon deel bawme, and the thridde part fevirfoie, and staumpe hem, and tempere hem with stale ale, and lete the sike drinke therof." In Devonshire the sciatica is termed bone-shave, and the same word signifies in Somerset an horny excrescence on the heel of an horse. ? A. S. sceorfa, *scabies*.

This word has puzzled the etymologists. The *New English Dictionary*, while saying that "the meaning of *shaw* does not appear," seems to take for granted that the first syllable of the word refers to "bone"; but this is more than doubtful if we note the equivalents from the *Catholicon* and the *Ortus*. In Lewis and Short's *Latin Dictionary* two references are given where *oscedo* signifies "a sore in the mouth of children, aphthæ."

It is only in the unsatisfactory state of the etymology of this word and with the utmost diffidence that I suggest that, as the word in its form of "baneschawe" originally described an aphthous condition of the mouth, its first syllable is "bane" in the sense of destruction or poison, and that the second syllable is (1) allied to "scall," which is defined in Todd's Johnson as

"a discontinuity of skin or flesh by a gnawing malady." Scaldhead is a term in common use at the present day. We know from the *Catholicon* that the word "scalde" in another sense was also spelled "scawde." Or (2) that it is a modification of "schavynge" in the sense of *abrasio* as given in the *Promptorium.* In either case, the word would then signify a specially troublesome ulcer. Possibly its corruption into "bone shave" followed from the confusion arising from "os" signifying both mouth and bone.

Fevirfoie mentioned in the prescription quoted by Mr. Way from the Sloan. MS. was the herb known also as feverfew, which was supposed to have what some still call "febrifuge" qualities.

**Chaucer's "Doctour of Phisyk."**—In the March number in an extract concerning the Black Death occurred some reference to Chaucer's celebrated "Doctour of Phisyk." Readers will, I have no doubt, be glad to have, accompanied by some notes which I have gleaned from various sources, the full description of that practitioner, especially as I can give a picture of him, taken from the coloured drawing in the Ellesmere MS., which is the best text of the *Canterbury Tales.* Dr. Furnivall kindly enabled me to obtain this from Messrs. Clay and Sons, who very courteously sent me in addition much that is of interest in connection with Chaucer. The doctor is thus introduced in the Prologue (ll. 411–44):

With us there was a Doctour of Phisyk,
In all this world ne was ther noon him lyk
To speke of phisik and of surgerye;
For he was grounded in astronomye.
He kepte his pacient a ful greet del
In houres, by his magik naturel.
Wel coude be fortunen the ascendent
Of his images for his pacient.
He knew the cause of everich maladye,
Were it of hoot or cold, or moiste or drye,
And where engendred, and of what humour;
He was a verrey parfit practisour.
The cause y-knowe, and of his harm the rote,
Anon he yaf the seke man his bote.
Ful redy hadde he his apothecaries,
To send him drogges, and his letuaries,
For ech of hem made other for to winne;
Hir frendschipe was nat newe to biginne.
Wel knew he the olde Esculapius,
And Deiscorides, and eek Rufus:
Old Ypocras, Haly, and Galien;
Serapion, Razis, and Avicen;
Averrois, Damascien, and Constantyn;
Bernard, and Gatesden, and Gilbertyn.
Of his diete mesurable was he,
For it was of no superfluitee,
But of greet norissing and digestible.
His studie was but litel on the Bible,
In sangwin and in pers he clad was al
Lyned with taffata and with sendal;
And yet he was but esy of dispence;
He kepte that he wan in pestilence.
For gold in phisik is a cordial,
Therfor he lovede gold in special.

From Chaucer's description of the medical pilgrim much may be learned, not only of the individual characteristics of this doctor, but of the contemporary state of medicine and its literature. Many of us might envy this practitioner's power of diagnosis and his etiological skill. Such a combination would go far to make "a verrey parfit practisour," as the advertising Australian doctor believed it did in his case, when he announced that his diagnosis was not only intuitive but instantaneous. The simplicity of the humoral pathology enabled the "Doctour of Phisyk" to define the cause of every malady with great precision.

The steed upon which the doctor is seated is not one that would satisfy the requirements of the dashing doctor of to-day. Neither of the artists of the Ellesmere MS. could draw a horse, for all the animals ridden by the pilgrims are of the same wooden description, as may be seen from the reproductions in the Chaucer Society edition of the Tales and the illustrated edition of Green's *Short History of the English People* (vol. i. pp. 420-9). The drawing in Urry's (1721) edition of Chaucer provides the doctor with a noble-looking animal, but the picture altogether lacks the interest and the character of that of the Ellesmere MS.

But in the matter of dress the doctor at the end of the nineteenth century, with his black morning or frock coat and dull ungraceful trousers, although he may have gained in comfort, is, so far as appearance goes, at a manifest disadvantage, not only with his fourteenth century ancestor, but with the doctor of later times, as may be seen from Allan Ramsay's portrait of Mead now in the National Portrait Gallery, or that of Astley Cooper by Sir Thomas Lawrence. Chaucer's doctor was arrayed "in cloth of a blood-red colour and of a blueish-grey." Todd says (*Illustrations of the Lives and Writings of Gower and Chaucer*, 1810, p. 254), "in the Manuscript his surcoat is of bright purple and his hood . . . of blue, deeply furred with white," and refers to a passage in *Piers the Plowman* (Passus VI.), written shortly before the *Canterbury Tales*, alluding to the benefits arising from an abstemious diet, and which contains a mention of the dress of a physician, and is also a good instance of the frequent satire directed against doctors, who are said to shorten rather than lengthen their patients' lives.

And if thow diete thee thus,
I dar legge myne eris
That Phisik shal hise furred hodes
For his fode selle,
And his cloke of Calabre,
With alle the knappes of golde,
And be fayne, by my feith!

His phisik to lete,
And lerne to laboure with lond,
For liflode is swete.
For murthereris are manye leches,
Lord hem amende!
They do men deye thorugh hir drynkes,
Er destynee it wolde.
ed. WRIGHT, 1856, vol. i., p. 133.

"Pers" in Chaucer's description is the obsolete French word for blueish-grey. Taffata and sendal, which formed the linings of the doctor's garments, were varieties of expensive silk; but though he allowed himself, probably for professional reasons, this extravagance of costume, he was exceedingly careful about his expenditure in other matters: "he was but esy of dispence." The "cloke of Calabre" in Langland is one made of a kind of fur, which is supposed, without any authority, to have derived its name from Calabria. The term, which is of frequent use in old literature, is now applied to the fur of the Siberian squirrel. Whitaker in his edition of *Piers* (p. 143) paraphrases the expression into "cloak of Salerno," perhaps thinking that as Calabria is in Italy the reference was to the School of Salernum.

The doctor is represented carrying a "urinal," and inspecting the secretion which it contains. Urinal was the name applied to the receptacle in which the urine was placed when the physician pretended, by a mere inspection, to diagnose the complaint from which a patient was suffering. The special marks which showed that Valentine was in love were so obvious, that Speed said to him, they "shine through you like the water in an urinal, that not an eye that sees you but is a physician to comment on your malady" (*Two Gentlemen of Verona*, ii. 1, 40–3). This piece of humbug was raised to the position of a fine art, for those who practised it would first show their ingenuity by learning indirectly what were the symptoms of the malady of the patient, and then achieve a reputation by declaring that the urine showed that the sick person had such symptoms. Not even the denunciations of Linacre, or a statute of the College of Physicians which declared that no one connected with that institution should upon such evidence prescribe for a patient whom he had not seen, could put a end to the practice which existed from at least 1230 through several centuries. Shakspere has several references to it; on these some comments were made in the *Journal* for December, 1887.

The way in which doctor and apothecary played into one another's hands does not need much comment. It survived long after Chaucer's time. A good illustration of it may be seen in Bullein's *Dialogue against the Fever Pestilence* (1578), from which an extract was given in this *Journal* for December, 1892. Since those days we have grown so good that of course no instance of the practice exists now.

The employment of astrology ("astronomye") in the treatment of the sick came into Western practice through the Arabians, and reached us, with many other evil things, by way of Italy. Many events of life, including the administration of remedies in illness, had, according to the astrologers, to be undertaken, if the result was to be successful, at an opportune moment, which was determined by consideration of the so-called planetary influences current at the time with those which ruled at the birth of the person concerned. No doubt the special love of gold with which Chaucer's doctor was possessed would account for the delay which occurred before he could "fortunen the ascendent" for his patient, or in other words declare that the lucky moment had arrived when there was a favourable influence of the ascending stars and planets. The "images" were the astrological designs by which the doctor represented the favourable aspect of the heavens. A drawing of one such image is given in *Good Words* for this month in an article by Sir Robert Ball on Kepler, who is a conspicuous example of a man with a scientific mind tainted with the absurdities of astrology. It might have been thought that such superstition had been outgrown now by all except the buyers of cheap almanacs, in which the pretension is made of foretelling future events; but we are told that astrologers are doing a good trade in England at the present day, and that the house of one of them "is visited by many leading people in society, while more than one of our commercial magnates and Stock Exchange speculators seek his advice" (*Review of Reviews*, March, 1893, p. 287). Only within the last few weeks I received the prospectus of a half-guinea book, which treats of natal astrology, and which contains chapters on "The Health of the Native" and "Diseases caused by the Planets." One of the authors of the book offers to supply "directions" for periods from one to fifty years, at prices ranging from 3/- to £5. Those who assert that the world is still in its childhood verily have some justification for the statement.

In the last line but one of the description is probably a reference to the "gold cure" of the middle ages. There was a widespread belief that gold taken internally would preserve youth and health, and heal all diseases. The writers of the time have many words about "aurum potabile" and its supposed virtue. Shakspere has one or two references to it (2 *Henry IV.*, iv. 4, 161–3; *All's Well that Ends Well*, v. 3, 101–4).

Chaucer's doctor was careful of his bodily welfare, and wisely enjoined for himself a very rigid and spare diet; but he seems to have been somewhat regardless of his spiritual growth.

The reading which had helped the doctor to become such an excellent practitioner was extensive, and derived from most of the nations which had contributed to the literature of medicine. He would have not had much difficulty in acquiring what there was to know of the old Æsculapius, for no literature is attributed to his name, and of his doings nothing is recorded except in a few notices in Greek Poetry. Although some writers believe him to have been an actual personage, he is usually regarded as the mythical son of Apollo and Coronis. His sons Machaon and Podalirius are well known, the former especially, as attending to the wounds of the combatants before Troy. Their services were highly valued. In the words of the version by one resident in Bristol:

> For more than a multitude availeth the leech for our need
> When the shaft sticketh deep in the flesh, when the healing salve must be spread.
> *The Iliad of Homer done into English Verse.* By ARTHUR S. WAY. 1877. XI. 514-5.

The deeds attributed to Æsculapius caused his name to be highly honoured. After-generations deified him and built temples in his honour. At these many wonderful cures were of course performed, and those who received benefit left behind them a description of their diseases, to which the priests of the temple added an account of the remedies employed. Those who practised the healing art took their methods from these records, represented themselves to be his descendants, and for some time were known as Asclepiadæ.

For some centuries medicine was largely in the hands of the philosophers, and it was not until 400 B.C. that Hippocrates became almost its first systematic practitioner. His writings were considerable and, characterised by wide powers of observation and a good style, had an enormous influence upon medical science. Several complete Greek and Latin editions of the works attributed to him were published in the sixteenth century, and these have been often reprinted. In the Bristol Medical Library will be found Latin versions dated 1620 and 1657, together with the Old Sydenham Society's well-known 1849 issue of his "genuine" writings, translated by Francis Adams. There have been countless commentaries on his works. Of sections of his writings the most frequently issued have been the "Aphorisms," of which there are editions in Greek, Latin, French, Spanish, English, Dutch, and German. We have Greek, Latin, and English versions bearing dates of 1623, 1684, 1735, 1736. We have also articles on Hippocrates by Warburton Begbie (*Brit. M. J.*, 1872, vol. ii.), Matthews Duncan (*Edin. M. J.*, vol. xxii., 1876), G. H. B. Macleod (*Brit. M. J.*, 1877, vol. ii.), and Finlayson (*Glasg. M. J.*, vol. xxxvii., 1892).

Dioscorides was a physician of Asia Minor about the time of Nero (54–67 A.D.). He paid special attention to Materia Medica, on which he wrote a treatise. This and his other works dealing principally with therapeutics were printed in Greek and Latin in the fifteenth century and afterwards. His editors, who have been numerous, have by their comments added much to his original writings. In the Bristol Medical Library there is a 1598 Latin copy of all his known works. The mediæval doctor, always an ardent therapeutist, was largely indebted to Dioscorides, who obtained an allegiance in the department of Materia Medica as great and lasting as did Galen in the sphere of general practice.

Towards the end of the first century of the Christian era, Rufus of Ephesus was distinguished more as an anatomist than a doctor. His work was highly spoken of by other writers, but as only fragments of it are extant it is not likely that Chaucer's doctor derived much assistance from it.

In the second century there was born at Pergamum, in Asia Minor, one who was destined to have a larger influence on matters medical than any other writer before him except Hippocrates, and the magic of whose name lasted like a spell over the healing art for centuries. Even in the reign of Elizabeth it was thought rank heresy to impugn his authority. (See *The Roll of the Royal College of Physicians of London*, 2nd ed., 1878, vol. 1, p. 62). Galenical or vegetable pharmacy was regarded almost as a sacred thing, and received no serious addition or opposition till Paracelsus at the beginning of the sixteenth century ventured to introduce mineral substances and chemical combinations into the Materia Medica. Galen was a voluminous author, and numerous large editions of his works, and commentaries on them, are in existence. We have in our library a five volume Greek version of his complete works, printed in 1538; and articles concerning Galen or his writings, by Kidd (*Tr. Prov. M. & S. Assoc.*, vol. vi., 1837), an anonymous writer (*Lond. M. G.*, 1844, vol. 1), Gasquet (*Brit. & For. M. Chir. Rev.*, vol. xl., 1867), Macleod (*Brit. M. J.*, 1877, vol. ii.), and Finlayson (*Brit. M. J.*, 1891, vol. ii.).

In the literature of the Doctor of Physic there is now a great chronological gap. In the year 792 begun the reign of the Caliph Haroun Al Raschid, who, following in the steps of his predecessors in encouraging the progress of literature, commissioned Mesue, who there is reason, from the title of one book, to believe was also known by the name of John Damascene, to translate Greek scientific works into his own language. He could not, however, have done much for the medical pilgrim to Canterbury; for although his own works are quoted by later writers, there is, except the one book to which I have referred, nothing that can with certainty be attributed to him.

There is much doubt as to the Serapion with whose writings Chaucer's doctor was acquainted. There was a Serapion of Alexandria whom Celsus mentions. The earlier of the two Arabian physicians of the same name lived at the beginning of the ninth century and was principally a compiler. His work found two Latin translators. The later Serapion, who died about 1070, left a work on Materia Medica, of which Latin editions were printed in the fifteenth and sixteenth centuries.

Rhazes, who was born in 852, was one of the most celebrated of the Arabian physicians. He had wide scientific knowledge, and a power, exceeding that of his predecessors, of presenting an orderly arrangement of ascertained facts which he accumulated in many works, accompanied by the record of his own observation of cases. He is now mainly known by his treatise on Small-pox, which, after going through several Latin and French editions from the end of the fifteenth century onwards, was translated into English by Mead in

1747. It was also issued by the Old Sydenham Society, translated from the original Arabic by Dr. Greenhill. Both these editions are in the Bristol Medical Library.

Haly, known better as Haly Abbas, was one of the leaders of Arabian medicine, and on account of his great learning was surnamed "Magus." In the latter half of the tenth century he wrote a comprehensive work dealing with the whole subject of medicine. It was translated into Latin in 1127, and was printed first in 1492 and again more than once in the sixteenth century.

Avicenna, whose name is more familiar perhaps than that of any other Arabian doctor, was born in 980. In early life he achieved a great reputation for proficiency in literature and mathematics, but seems to have wasted his great abilities by frivolities of life, and in almost entirely compiling from previous writers. Yet his works, especially his *Canon Medicinæ*, had a great influence upon his successors, and were authorities in medicine till quite the middle of the seventeenth century. They were first printed in Arabic, and afterwards in Latin.

Having given so much attention to medicine as expounded by the Arabs, it was to be expected that the Chaucerian doctor would bestow some study on the writings of the man who did most to introduce Arabian medical thought and practice into Europe. Constantinus, surnamed Africanus from the fact that he was born in Carthage, who had spent much of his life in the East, became in the latter half of the eleventh century identified with the School of Salernum, which was just then rising into prominence as a centre of medical teaching of university character. It exercised a long influence over medical life in general, and lived till 1811. In its corporate capacity it issued several works, the principal of which were a cyclopædia of medicine and surgery and the well-known *Schola Salernitana*, a dissertation on the preservation of health issued as a poem in Latin verse, and which enjoyed a popularity for centuries. It was first printed in 1474. Salernum is also celebrated for having produced several women doctors. Chaucer refers again to Constantine in "The Merchant's Tale," where an old man is represented as having taken freely of aphrodisiacs

> And many a letuary had he ful fyn,
> Such as the cursed monk daun Constantin
> Hath writen in his book *de Coitu*.

Constantine's complete works were printed in Latin in 1536.

The doctor in the *Canterbury Tales* had a great fancy for Arabian literature. Averrhoes, who added little to the store of medical knowledge, was a man of great intellectual attainments; he was more a theoretical than a practical physician. He lived in the second half of the twelfth century. Some of his medical works were translated into Latin and afterwards printed; but he is principally famous for his philosophic treatises.

Montpellier became almost as famous as Salernum as a school of medicine; and in the person of Bernard de Gordon supplied an authority for Chaucer's companion. Bernard was teaching there from 1285 to 1307. His most famous book was *Lilium Medicinæ*. His collected works were first printed in 1487.

Gilbertus Anglicus, whose date is not exactly known, but which was about the end of the thirteenth or beginning of the fourteenth century, was the first English author whose works have survived till the present day. Among them is a Compendium of Medicine, which was first printed in Latin in 1510. He is said to have derived his inspiration mainly from the Salernian school.

About 1280 was born a physician who Dr. Norman Moore *(Dict. Nat. Biog.)* thinks may have been the man whom Chaucer had in his mind's eye when he described his Canterbury pilgrim, who the Host considered was "a propre man and y-lik a prelat." He became known as John of Gaddesden, and went to Merton College, Oxford. As Chaucer was page in the household of Lionel, son of Edward III., and as Gaddesden attended some part of the Royal family, it is probable that they saw one another. About 1307 Gaddesden wrote his famous book, *Rosa Medicinæ*, of which there still exist many MSS. It was printed in 1492, and more than once in the fifteenth century. He died in 1361, having held a prebendal stall in St. Paul's. At that time Chaucer was probably about 21. Dr. Freind's account of Gaddesden (*Hist. Physic*, II. 277–91) shows that he was a very shrewd practitioner, but not one of whom we can be at all proud.

## Figures and Tables

**Figure f1:**